# The potential impact of ChatGPT in clinical and translational medicine

**DOI:** 10.1002/ctm2.1216

**Published:** 2023-03-01

**Authors:** Vivian Weiwen Xue, Pinggui Lei, William C. Cho

**Affiliations:** ^1^ Guangdong Provincial Key Laboratory of Regional Immunity and Diseases, Department of Pharmacology Carson International Cancer Center, Shenzhen University Health Science Center Shenzhen Guangdong China; ^2^ Department of Radiology The Affiliated Hospital of Guizhou Medical University Guiyang Guizhou China; ^3^ School of Public Health Guizhou Medical University Guiyang Guizhou China; ^4^ Department of Clinical Oncology Queen Elizabeth Hospital Hong Kong SAR China

1

The Chat Generative Pre‐trained Transformer (ChatGPT) is an artificial intelligence (AI) model developed by Open AI for generating human‐like text. ChatGPT is powered by the large language model (LLM) GPT‐3.5. Like other LLM‐based AIs, it has been trained on large datasets of text and can generate new text similar to the text it was trained on, which requires generating, understanding and interpreting human language using computer systems.^1^, ^2^, ^3^ ChatGPT simulates human interaction, and it is probably the most powerful language model currently available. It uses a deep learning technique called a ‘transformer architecture’ that is trained on massive text datasets obtained from the internet.^4^

ChatGPT is leading a revolution in AI technology, and its impact on the field of clinical medicine is yet to be determined. Some clinical practices may rely on data analysis, clinical research and guidelines. AI models may help in clinical decision support, clinical trial recruitment, clinical data management, research support, patient education and other fields.^5^, ^6^ In some cases, AI models can automate certain tasks performed by humans, such as data analysis, image acquisition and interpretation.^7^, ^8^ This may increase the efficiency and reduce the workload of healthcare professionals, allowing them to focus on higher level tasks that require their expertise and clinical judgement. On the other hand, the results must originate from the extraordinary human mind and its ability to process information and communicate through them.^9^ Therefore, it is important to use AI models such as ChatGPT as a tool to support, rather than replace, healthcare professionals in their decision‐making process.

Similarly, AI technology including ChatGPT also has great potential in assisting basic research and accelerating the technological transformation of clinical and translational medicine. For example, in terms of drug discovery, AI has image recognition capabilities, which can identify, classify and describe chemical formulas or molecular structures to assist the design of new structures and functional group combinations of compounds.^10^ In addition, AI technologies play an important role in disease prediction, diagnosis and assessment of therapeutic targets, such as providing treatment guidelines for cancer patients based on their magnetic resonance imaging radiomics and predicting ageing‐related diseases.^11^, ^12^, ^13^ However, unlike the AI algorithm or model specially developed for drug discovery and disease diagnosis, the core value and advantage of ChatGPT lies in its powerful LLM. At present, ChatGPT cannot update the training data in a real‐time manner. In addition, it can only give general and vague answers in some existing medical‐related conversations.^14^ Some experts present conversations with ChatGPT online for case studies and they find that the diagnoses made by ChatGPT are often not comprehensive and adequate. For example, for patients with common symptoms such as fever, ChatGPT will give suggestions to take antipyretics to help relieve symptoms, but cannot accurately judge infection, pimples or other causes. Therefore, blindly relying on the diagnosis and guidance of ChatGPT may have the potential risk of inaccurate diagnosis or delayed treatment. Furthermore, Fijačko et al. pointed out that ChatGPT without specific courses or training in medical knowledge could not pass the life‐support exam, suggesting that ChatGPT may not be capable enough for life support in clinical applications.^15^ This evidence implies that ChatGPT may not be capable of independently handling the complex work of clinical practice in the current version.

Therefore, focusing on the application of ChatGPT in human–computer interaction may improve its usability in clinical practice. For example, the diagnosis and treatment of mental illness rely heavily on doctor–patient questionnaires, interviews and judgement. However, many interfering factors such as the physician's tone of voice, mood and the surrounding environment can hinder an accurate assessment of the disease. In this field, AI has been used to record and analyze data related to questionnaires.^16^ The emergence of ChatGPT may accelerate the integration of flexible questionnaires, documentation, diagnosis and follow‐up of patients with mental disorders using chatbots. In addition, ChatGPT provides a basis for more flexible and efficient epidemiological research. Indeed, epidemiological research also relies on efficient and reliable data collection, recording and analysis.^17^ ChatGPT not only solves the difficulty of remote inquiries, but also helps to reduce labour requirements to complete the work. Besides, ChatGPT is usually more accurate and faster than manual statistics and records.

ChatGPT has caused some changes and controversies in a short period of time in terms of medical education, training and writing.^18^, ^19^ It has been used to finish writing an abstract, introduction or even the main text of an assignment or article, but it may not do well in writing the creative part of medical writing.^20^ ChatGPT often summarizes previous research and data to form abstracts or background knowledge. However, some issues, including ethical concerns, need further clarification.^21^ Some scientific journals noted that articles containing ChatGPT‐generated content need to be explicitly listed as authors, while *Nature* refused to accept ChatGPT as an author on articles because it cannot be responsible for content generated by itself.^22^

Furthermore, AI models including ChatGPT can clearly help the healthcare industry by providing a more objective and evidence‐based approach to decision‐making, reducing the risk of human error based on its unparalleled speed of information processing. It also helps identify patterns and correlations in vast amounts of data, which may provide new insights and discoveries in medicine. Additionally, AI models can assist in the detection of disease and the prediction of prognosis,^23^ which may help provide more personalized treatment recommendations and improve patient outcomes.^24^ Further research and development are needed to ensure the effective and responsible use of AI models in the healthcare industry. Nevertheless, we should be aware that ChatGPT is a double‐edged sword, with both powerful features and potential shortcomings.^3^ AI has the potential to significantly impact clinical and translational medicine by improving data analysis,^7^ streamlining workflow^25^ and enhancing decision‐making.^26^ However, potential negative impacts such as privacy concerns,^27^ bias, discrimination^28^ and so forth should not be underestimated.

Overall, ChatGPT has the potential to revolutionize clinical and translational medicine, but we need to develop appropriate strategies to mitigate potential risks and negative outcomes. Despite our initial lack of preparation for the game‐changing ChatGPT technology, the development of AI is unstoppable. The best course of action is to embrace it, use its capabilities to improve our lives, and foster mutually beneficial relationships by evolving it in clinical medicine.
